# Development and Validation of a Knowledge, Attitudes and Practices Questionnaire on COVID-19 (KAP COVID-19)

**DOI:** 10.3390/ijerph18147493

**Published:** 2021-07-14

**Authors:** Da-In Park

**Affiliations:** Department of Nursing, College of Life Science and Nano Technology, Hannam University, Daejeon 306-791, Korea; dpark@hnu.kr

**Keywords:** COVID-19, knowledge, attitudes, practice, instrument development and validation

## Abstract

Given its highly contagious nature and an absence of a specific antiviral agent to this date, the key to controlling the spread of coronavirus disease 2019 (COVID-19) and decreasing the infection rate is adherence to preventive measures. It is essential to understand an individual’s knowledge, attitudes and practices toward COVID-19 since public adherence to health guidelines relies heavily on these aspects. However, there is no validated instrument that evaluates knowledge, attitudes and practices toward COVID-19. Thus, this study aimed to develop and validate such tool. A questionnaire was developed based on international and national guidelines and a review of the literature. Initial items were evaluated by 10 experts to determine content validity. Exploratory factor analysis and reliability testing were conducted with a convenience sample of 229 nursing students. Based on the content, face validity and factor analysis, 34 items were selected. The Kaiser-Meyer-Olkin value of 0.735 indicated a highly acceptable score with a significant Bartlett’s test of sphericity (*p* < 0.0001). The internal consistency coefficients indicated acceptable reliability of the tool (Cronbach’s α = 0.75). The KAP COVID-19 is a valid instrument that can be used to evaluate knowledge, attitudes and practices toward COVID-19.

## 1. Introduction

The coronavirus disease 2019 (COVID-19) has spread around the world at an unprecedented speed. It is a highly contagious disease caused by coronavirus-2, also known as SARS-CoV-2. The COVID-19 was declared a global pandemic by the World Health Organization (WHO) in March, 2020 and has created public health concerns and threatened the economy worldwide [1]. Within a one-year period since the start of the pandemic, the cumulative confirmed cases have yielded over 98.2 million with over 2.1 million deaths around the globe within [2]. Human transmission of COVID-19 is reported to occur when infected respiratory droplets are expelled during close face-to-face contact while talking, sneezing or coughing [1,3]. Various symptoms have been reported, ranging from mild symptoms to severe complications such as acute respiratory distress syndrome, sepsis, hyperinflammatory response, multiorgan failure, thromboembolism and vascular damage [1]. Although vaccination against COVID-19 has recently started, no specific treatment is available for COVID-19 to this date [4]. The management of confirmed cases is continuously evolving based on expert opinions and guidelines, and they are primarily being treated based on pathological features, symptoms and for supportive care [5]. Therefore, governmental bodies have responded with containment measures, including national lockdowns, quarantines, curfews and social distancing, to prevent community transmission. Also, they have emphasized the importance of personal hygiene, including wearing face masks and handwashing.

Since the first reported confirmed case, the infection rate in Korea has rapidly increased to 70,000 over a one-year span [6]. Although various policies and interventions have been reinforced to safely and efficiently prevent, diagnose and manage COVID-19, the battle against the outbreak is still ongoing in Korea. Given its highly contagious nature and an absence of a specific antiviral agent to this date, the success or failure in controlling the spread of the disease and reducing the infection rates largely relies on public behaviors. The key to successfully controlling the spread of a virus relies on the individual’s preventive measures and adherence to public guidance. Studies have indicated that public adherence to disease prevention is largely affected by levels of knowledge, attitudes and practices (KAP) [7,8]. Past studies conducted during the 2002–2004 severe acute respiratory syndrome (SARS) outbreak and the 2012 Middle East respiratory syndrome (MERS) outbreak have suggested that an individual’s level of anxiety, stress, panic emotions and coping skills are significantly associated with knowledge and attitudes toward infectious diseases [7]. Furthermore, levels of knowledge and attitudes were found to affect individual attempts and efforts to prevent the spread of the disease.

Since the start of the global pandemic, a few studies have been conducted to investigate the KAP on the novel COVID-19 among different populations. These studies have allowed us to understand and identify those who are more likely to have negative attitudes and potentially dangerous practices toward the infectious disease. However, none of the studies have used a reliable and valid instrument [9,10,11,12]. All rigorous research designs should incorporate psychometrically sound measurement tools, and, therefore, the use of an instrument that lacks evidence of validity and reliability is considered critically problematic and decreases the strength of study findings [13,14]. Moreover, some of the previous studies only partially captured the elements of KAP [9,15]. A reliable and valid instrument that fully measures KAP on COVID-19 should be established to provide vital information in deciding the best intervention programs to change public misunderstanding about the disease and increase adherence to safety measures.

To facilitate successful and effective management of the COVID-19 outbreak, international and governmental agencies have advised all healthcare personnel to keep themselves updated on the infectious disease and to comply with standard guidelines and droplet precautions at all times [2,6]. These advisories are not only applicable to medical staff but also to students who are involved with clinical practicum in hospital settings. Nursing students spend a considerably large amount of time within the hospital for clinical hours and keep in close contact with patients. Therefore, it is necessary to investigate KAP toward the infectious disease among nursing students using a psychometrically sound instrument. Former studies on COVD-19 KAP that were mentioned only included adults, children and ethnic minorities, and there is a lack of studies focusing on the nursing student population [9,10,11,12].

Thus, this methodological study with cross-sectional data collection aimed to construct a new instrument that allows the assessment of knowledge, attitudes and practices on COVID-19 and to evaluate its psychometric properties in a sample of nursing students.

## 2. Materials and Methods

**Figure 1:**
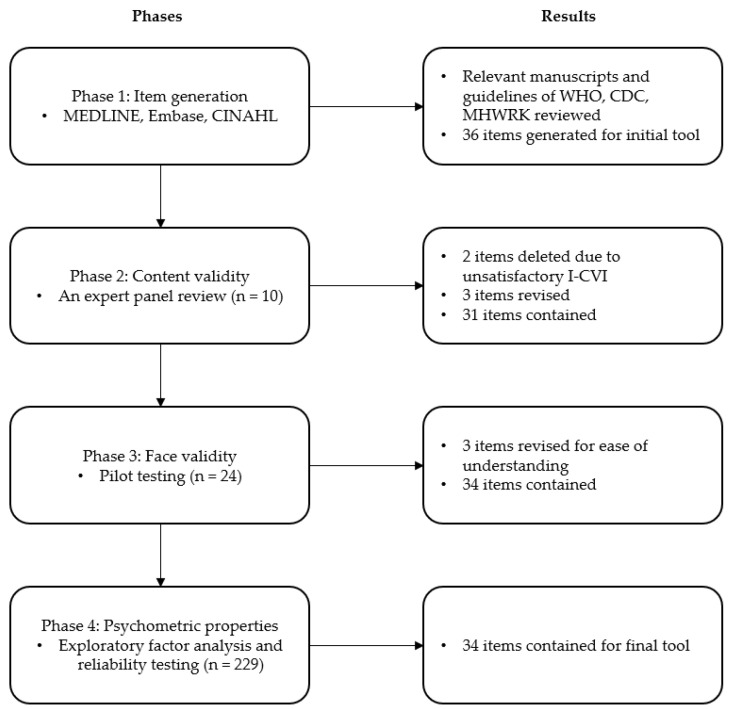
A flow diagram of the development and validation process.

The process of instrument development and validation of items for the KAP COVID-19 was undertaken in the following four phases: Phase 1, generating items that suit the study purposes by reviewing the relevant literature; Phase 2, evaluating content validity by sending the first draft of the instrument to a panel of expert reviewers and modifying the first draft according to the expert review; Phase 3, assessing face validity by conducting a pilot test of the modified instrument; and Phase 4, further modifying the instrument based on the results of exploratory factor analysis (EFA), examining construct validation and reliability of the final version of the tool (Figure 1).

### 2.1. Phase 1: Item Generation

First, a literature review was performed to define the construct of interest and to obtain the initial questionnaire. Searches of the literature were performed on several databases, including MEDLINE, Embase and CINAHL databases, using selected keywords, e.g., ‘coronavirus’, ‘coronavirus infections’, ‘COVID-19’, ‘health knowledge, attitudes, practice’, ‘infection control’ and ‘disease outbreaks’. Moreover, the guidelines and latest updates for clinical and community management of COVID-19 by the WHO, CDC and the Ministry of Health and Welfare of the Republic of Korea (MHWRK) were reviewed and analyzed for quantitative content. After the literature review, a total of 36 items were generated for the initial questionnaire.

### 2.2. Phase 2: Content Validity

To ensure the legitimacy of the preliminary questionnaire’s content, content validity indices (CVIs) were calculated for both the individual items (I-CVI) and the entire scale (S-CVI) by using the Delphi method [14,16]. It is recommended to include a minimum of six individuals for an expert panel to allow for at least one disagreement between experts [17]. Thus, a CVI tool and the first draft of the KAP COVID-19 instrument were sent to 15 expert reviewers who were carefully chosen based on their expertise areas in academia, instrument development research and healthcare. A heterogeneous panel of 10 experts voluntarily agreed to participate and returned completed Delphi surveys. Each expert was asked to rate each questionnaire item based on the relevance of the item content to COVID-19. The expert panel included three medical doctors, five registered nurses and two nursing professors. The medical doctors were active governmental COVID-19 epidemiological investigators, who have received several trainings from the Korea Centers for Disease Control and Prevention. Registered nurses had a minimum of 5 years of clinical experience and were working full-time at nationally designated COVID-19 treatment facilities throughout the pandemic. Two nursing professors taught fundamental and clinical practicum courses during the pandemic and had a research background in instrument development. The experts were asked to evaluate how well each item corresponds or reflects a specific domain on a four-point Likert scale. The scoring method was as follows: 1 = not relevant, 2 = somewhat relevant, 3 = relevant and 4 = highly relevant [16]. They were also invited to give comments regarding each item and the general formulations of the initial questionnaire.

The content validity of the initial KAP COVID-19 was assessed using the CVI, which adds clarity when judging the degree to which tool items are relevant to the concept being assessed [17]. Items with an I-CVI value of ≥0.78 were retained, those with I-CVI between 0.70 and 0.78 were revised, and those with I-CVI ≤0.70 were excluded [16]. After the I-CVI calculation, the scale-level content validity index was analyzed using both the average method (S-CVI/Ave) and the universal agreement method (S-CVI/UA). In addition, the expert panel’s recommendations were taken into consideration in revising some of the wording and phrasing of items.

### 2.3. Phase 3: Face Validity

A pilot test was conducted to assess the face validity of the tool to obtain feedback from a convenience sample of nursing students. Those who met the inclusion criteria and gave informed consent voluntarily were included. They were asked to answer each and every item, to comment on the ease of understanding each item and to identify any ambiguous words or phrases. Moreover, the average length of time used to complete the questionnaire was assessed.

### 2.4. Phase 4: Psychometric Analysis

To determine the psychometric properties of the KAP COVID-19, including construct validity and internal consistency reliability, a larger number of nursing students were invited to complete the tool. The study design, inclusion and exclusion criteria and the data collection procedure were kept exactly the same as the pilot test. Data were collected for the main study in assessing psychometric properties from October to December 2020. Collected data were used to analyze an exploratory factor analysis and reliability test.

First, intercorrelations between variables were tested using Field’s recommendation [18]. Items with a bivariate correlation score of >0.80 were removed. Then, the Kaiser-Meyer-Olkin (KMO) test and Bartlett’s test of sphericity were assessed for sampling adequacy [14]. These two tests allow an assessment of the suitability of conducting a factor analysis for the given data or the sampling adequacy [14]. Data factorability was determined by a KMO value greater than 0.50 and significance of Bartlett’s test of sphericity value (*p* < 0.05) [14]. Once factorability was confirmed, a principal component factor analysis using orthogonal rotation, a method of rotation that provides a clear and more interpretable structure by explaining variances among the factors that do not overlap and are independent from each other, was performed [14,19]. Eigenvalue, scree plot and variance were analyzed to determine the number of factors. EFA with a cut-off point of 0.40 for factor loading for the extraction of scale dimensions was performed [14,20]. Each extracted factor was given a meaningful name after carefully examining all items in each factor.

Internal consistency reliability was evaluated using Kuder-Richardson-20 (KR-20) for the dichotomous scale (knowledge subscale) and Cronbach’s α coefficient for the four-point Likert scale (attitude and practices subscales). KR-20 coefficient of ≥0.50 and Cronbach’s α coefficient of ≥0.70 were considered satisfactory evidence of internal consistency for the new instrument [14,21,22].

### 2.5. Sample and Data Collection

After item generation and content validation, a convenient sample of nursing students at a university located in Korea was recruited. The inclusion criteria for both the pilot and main studies were that participants were at least 18 years of age, were enrolled in an undergraduate nursing program and were full-time students during the academic year of 2020 after the COVID-19 pandemic breakout. Although there is no gold standard, the recommended sample size to validate a newly developed instrument is a minimum of five individuals per item [23,24]. Therefore, at least 170 participants were required for the 34-item KAP COVID-19. The final sample of the main study consisted of 229 participants.

### 2.6. Statistical Analysis

Statistical analysis was performed using IBM^®^ SPSS software version 23.0 (SPSS Inc, Chicago, IL, USA) with a significant value of *p* set at <0.05. Demographic characteristics were described with descriptive statistics, such as means for continuous variables and frequency for categorical variables, as appropriate. As for the expert panel evaluation, Microsoft Excel was used to analyze the content validity. All data analyses were conducted based on the previously mentioned criteria.

### 2.7. Ethical Consideration

The study protocol was reviewed and approved by the institutional review board (IRB No. 20-03-02-1020). Potential study participants were provided with a detailed description of the study and were assured of confidentiality. Written, informed consent was obtained from each participant. They were also informed of the voluntary nature of the study participation and completion without any negative consequences.

## 3. Results

### 3.1. Demographic Characteristics

#### 3.1.1. Pilot Test

The pilot test was conducted on a group of 24 female nursing students. The mean age of the respondents was 21.42 ± 1.56, and they were mostly third-year students (N = 13, 54.2%).

#### 3.1.2. Main Study

A total of 229 full-time nursing students participated in the main study. On average, the participants were 21.1 ± 1.7 years of age and predominantly female (91.7%), and the majority of the students (71.2%) were enrolled in clinical practicum courses during the COVID-19 pandemic. The mean score of the total KAP score was 83.1 ± 11.1 (Table 1).

### 3.2. Phase 1: Item Generation

In the preliminary phase, 39 items were extracted from reviewing the relevant literature and guidelines. Then, items were organized into three subscales with sub-domains. After removing three repetitive items, the first draft of KAP COVID-19 included 36 items (15 items on knowledge, 7 items on attitudes and 14 items on practices).

### 3.3. Phase 2: Content Validity

Based on the comments of the expert panel, two items with I-CVI values of 0.40 and 0.60 were excluded, three items were revised and 31 items were retained for construct validation (Table 2). After excluding two items with unsatisfactory I-CVI, the S-CVI/Ave and S-CVI/UA values of the 34 items were 0.96 and 0.65, respectively.

### 3.4. Phase 3: Face Validity

Regarding the phrasing and wording of the KAP COVID-19, only a few minor issues were reported during the pilot study. The participants commented that they had difficulties with some of the items and had to read more than once to fully understand. Therefore, minor revisions were made during Phase 3. For instance, item 4 (item 3 in the first version) was revised from “COVID-19 always cause severe acute respiratory complications” to “All confirmed cases of COVID-19 develop severe acute respiratory complications” for clarity. Moreover, “with unwashed hands” was added to item 12 (item 14 in the first version), “As a precautionary measure, one should avoid touching eyes, nose and mouth,” and item 25 (item 27 in the first version), “I consciously avoid touching my eyes, nose and mouth as much as possible.” All respondents took less than 15 min to answer and comment on all items.

### 3.5. Phase 4: Psychometric Properties

After the final version of 34-item KAP COVID-19 was produced, the questionnaire was distributed to the 229 main study participants to test its psychometric properties. Based on the EFA results of the collected data, each item with a >0.40 loading value was assigned to an extracted factor.

The correlation matrix indicated an acceptable correlation lower than 0.80. The KMO value of 0.735 indicated that the data were suitable for factor analysis, and the Bartlett’s test of sphericity was significant (*p* < 0.0001). The scree plot indicated 10-factor solutions, and all of the item’s extracted commonalities were indicated as acceptable. Further analyses were performed on each subscale of the tool to confirm extracted factor solutions. An adequate KMO value and significant Bartlett’s test of sphericity for the knowledge subscale (0.57, χ^2^ = 314.32, *p* < 0.001) and a six-factor structure solution with eigenvalues greater than 1.0 were extracted with 65.57% of the total variance explained. The extracted dimensions were the following: nature of the disease, signs and symptoms, diagnosis, medical management, patient under investigation management and quarantine, and precautionary strategy. The attitudes subscale showed satisfactory KMO and Bartlett’s test of sphericity values (0.68, χ^2^ = 731.66, *p* < 0.001). Two components were extracted with 67.17% of the total variance. The two identified components were “beliefs about COVID-19 prevention” and “beliefs about healthcare provider role”. The satisfactory KMO value of 0.87 and Bartlett’s sphericity value of <0.001 for the practices subscale indicated factorability of the items. The scree plot indicated a two-factor structure with 47.52% (Table 2). Two dimensions include concepts of “personal hygiene practices” and “restricting behavior.” All factor loadings were significant with values greater than 0.40, indicating an adequate proportion of common variance among the items in each scale. The factor loading values and items loaded into each identified EFA dimension are shown in Table 3.

The internal consistency reliability of the factored 34-item scale was obtained. The overall Cronbach’s alpha vale for the 34-item scale indicated an acceptable reliability (α = 0.75). The KR-20 coefficient for the knowledge subscale implied adequate internal consistency with a value of 0.53. Cronbach’s α coefficient of both attitudes (0.72) and practices (0.85) subscales also indicated satisfactory internal consistency.

## 4. Discussion

This study developed and demonstrated the psychometric evaluation of KAP COVID-19 in a sample of nursing students. The psychometric assessment provided sound evidence for its validity and reliability in evaluating knowledge, attitudes and practices toward COVID-19. Our results showed acceptable content, construct validities and reliability.

The KAP COVID-19 questionnaire consists of 34 items and contains knowledge, attitudes and practices subscales. The initial questionnaire was developed based on an extensive literature review of research and global and national guidelines. Then, the content analysis was conducted to exclude items that did not represent the complete range of the attributes under the study. In this study, an expert panel consisting of 10 experts with various experiences related to COVID-19 was formed to avoid an inflated estimate of validity. Moreover, construct analysis and reliability testing were conducted to identify structural dimensions and determine the internal consistency. Although the reliability of the 13 items pertaining to knowledge had a relatively lower KR-20 coefficient value than expected, the items should remain, as they were derived from a rigorous literature review as well as recommendations from the expert panel. Moreover, similar questions pertaining to knowledge were included in previous studies that were conducted among various populations, including medical students, healthcare workers and the general public [7,11,25].

This is the first study to rigorously develop and investigate the psychometric properties of the KAP COVID-19 instrument. Content and construct validation methods and reliability testing using exploratory factor analysis were conducted. Moreover, the relevant scientific literature and the guidelines of the World Health Organization, the Centers for Disease Control and Prevention and the Ministry of Health and Welfare of the Republic of Korea were used to determine and generate items and domains of the scales. This study has used an extensive literature review and expert suggestions for tool development and provides evidence for the psychometric properties of the KAP COVID-19. Therefore, its use is recommended to assess knowledge, attitudes and practices before and after training courses on the disease and in other related research.

A few limitations should be taken into consideration in interpreting the results of this study. First, considering the large proportion of the participants being female, it may not represent the whole nursing student population. Second, the test–retest reliability, which examines the temporal stability of an instrument over time, was not analyzed in this study due to its cross-sectional design [14]. Nonetheless, further studies are recommended to verify the stability of KAP COVID-19 in various populations.

## 5. Conclusions

This study has demonstrated the KAP COVID-19 to be a 34-item multidimensional scale with robust psychometric properties. It is a valid instrument that allows an assessment of knowledge, attitudes and practices levels contributing to adherence to guidelines against infectious disease control. Study findings provide support for the satisfactory reliability and validity of the KAP COVID-19. Despite the limitations mentioned above, this newly developed instrument is a measure that could prove its use for a better understanding of current knowledge, attitudes and practices. The use of this tool may help policymakers, health educators, clinicians and researchers identify the target populations for COVID-19 prevention and education.

## Figures and Tables

**Table 1:** Characteristics of study participants.

Characteristics	(*N* = 229)
Age (years), M ± SD (range)	21.1 ± 1.7 (18–33)
Gender, n (%)	
Female	210 (91.7)
Male	19 (8.3)
Grade, n (%)	
Freshman	26 (11.4)
Sophomore	40 (17.5)
Junior	95 (41.5)
Senior	68 (29.7)
KAP COVID-19	
Knowledge scale, M ± SD (range)	8.9 ± 2.0 (3–13)
Attitude scale, M ± SD (range)	32.8 ± 6.9 (16–46)
Practices scale M ± SD (range)	41.4 ± 7.7 (19–56)
Total score, M ± SD (range)	83.1 ± 11.1 (50–110)

KAP: Knowledge, Attitudes and Practices.

**Table 2:** Content validity index (CVI) rating of the individual KAP COVID-19 items.

Item	I-CVI
**Knowledge subscale: Level of knowledge on COVID-19 symptoms, diagnosis, treatment and transmission**	
1. COVID-19 is a respiratory syndrome caused by SARS-CoV2 virus infection	1.0
2. The SARS-CoV-2 is an RNA virus belonging to Coronaviridae family	1.0
3. COVID-19 is classified as a first-class infectious disease	0.4 ^†^
4. Other than the main clinical symptoms of COVID-19, patients may also experience sore throat, headache, nausea and diarrhea	0.8
5. COVID-19 always cause severe acute respiratory complications	0.8
6. COVID-19 is currently being diagnosed using sequencing method	0.8
7. COVID-19 is currently being diagnosed using a real-time reverse transcriptase polymerase chain reaction (Real-Time PCR) test	0.9
8. Current treatment for COVID-19 is symptomatic treatment (treating the symptoms of a disease) including fluid supplementation, administration of antipyretic drugs, etc.	1.0 ^‡^
9. A targeted therapy is available for SARS-CoV-2	0.9
10. To this date, there is no antiviral agent specific to SARS-CoV-2	1.0
11. COVID-19 is spread through droplet transmission	0.5 ^†^
12. If a symptomatic patient tests negative for SARS-CoV-2, he/she must follow self-quarantine guidelines (i.e., remain at home, stay away from others including family members, etc.) for 14 days after the onset of symptoms	0.8 ^‡^
13. Medical treatments and self-quarantine are not required if suspected patients (i.e., individuals displaying clinical symptoms within 14 days of contact with confirmed COVID-19 patients) test negative on PCR tests	0.9 ^‡^
14. As a precautionary measure, one should avoid touching eyes, nose and mouth	1.0
15. As a precautionary measure, surrounding environment should be disinfected and ventilated frequently	1.0
**Attitudes subscale: The level of attitude towards the pandemic crisis and healthcare system**	
16. I believe that COVID-19 can be prevented if I follow Korean government’s guidelines	1.0
17. I believe that COVID-19 can be prevented if I follow WHO guidelines	0.9
18. I believe that COVID-19 can be prevented if I follow CDC guidelines	0.8
19. Healthcare providers’ active participation in hospital infection control guidelines can reduce the spread of COVID-19	1.0
20. All pertinent information about COVID-19 should be shared among healthcare providers	1.0
21. Healthcare providers should be aware of all pertinent information about COVID-19	0.8
22. Confirmed COVID-19 patients should receive immediate and prompt medical care	1.0
**Practices subscale: The level of self-protecting practices during the pandemic crisis**	
23. I wash my hands more frequently than usual	1.0
24. I try to thoroughly wash my hands with soap and water for at least 30 s	1.0
25. I use hand sanitizers more frequently than usual	1.0
26. I consciously cover my nose and mouth with tissue, handkerchief or cloth when I cough or sneeze	1.0
27. I consciously avoid touching my eyes, nose and mouth as much as possible.	1.0
28. I minimize and avoid using public transportation as much as possible	0.9
29. I feel reluctant to use public transportation	0.9
30. I avoid going out as much as possible	0.8
31. I avoid crowded places (e.g., restaurants, department stores, shopping malls, bars, clubs, etc.) as much as possible	0.9
32. I have cancelled or postponed personal and social activities such as meeting, gathering, dining out, shopping, travelling with family, friends and acquaintances to reduce contacting other people	1.0
33. I avoid physical contact (e.g., handshakes, hugs, etc.) with others as much as possible, and maintain at least 2 m physical distance with others	0.9
34. If I feel unwell, I avoid going out and stay home for 3~4 days	1.0
35. Other than clinical practicum hours, I avoid visiting hospitals or pharmacies as much as possible	0.8
36. I avoid walking around the neighborhood or visiting other neighborhood areas	0.7

^†^ deleted item; ^‡^ revised item.

**Table 3:** Results of the exploratory factor analysis.

Factor	Item	Factor Loading ^a^	Eigenvalue	VE (%)	CVE (%)	Cronbach’s α
**Knowledge subscale**						0.532 ^b^
Nature of the disease	1	0.781	4.219	12.409	12.409	
	2	0.534				
Signs and symptoms	3	0.744	2.693	7.922	20.331	
	4	0.651				
Diagnosis	5	0.657	2.545	7.485	27.816	
	6	0.854				
Medical management	7	0.554	2.235	6.575	34.391	
	8	0.677				
	9	0.742				
PUI management and quarantine	10	0.751	1.678	4.963	39.326	
	11	0.823				
Precautionary strategy	12	0.851	1.585	4.663	43.989	
	13	0.820				
**Attitudes subscale**						0.720
Beliefs about COVID-19 prevention	14	0.897	1.541	4.531	48.521	
	15	0.963				
	16	0.901				
Beliefs about healthcare provider role	17	0.496	1.481	4.355	52.875	
	18	0.810				
	19	0.731				
	20	0.701				
**Practices subscale**						0.851
Personal hygiene practices	21	0.716	1.400	4.117	56.992	
	22	0.748				
	23	0.593				
	24	0.605				
	25	0.561				
Restricting behavior	26	0.587	1.296	3.811	60.803	
	27	0.512				
	28	0.778				
	29	0.781				
	30	0.651				
	31	0.680				
	32	0.517				
	33	0.621				
	34	0.694				

VE, variance explained; CVE, cumulative variance explained; PUI, person under investigation. ^a^ Extraction method: principal component analysis. Rotation method: varimax with Kaiser normalization. Factor loading cut-off: >0.40. ^b^ Kuder-Richardson-20 coefficient.

## Data Availability

Data available on request.
